# Methyl 4-(4-fluoro­phen­yl)-6-isopropyl-2-(methylsulfonyl)pyrimidine-5-carbox­ylate

**DOI:** 10.1107/S1600536808013536

**Published:** 2008-05-21

**Authors:** Wei He, Hong-Shun Sun, Chun-Xiang Ji, Dong-Ling Yang, Cheng Guo

**Affiliations:** a Department of Applied Chemistry, College of Science, Nanjing University of Technolgy, Xinmofan Road No. 5, Nanjing 210009, People’s Republic of China; b Nanjing Frochem Tech. Co. Ltd, Xinmofan Road No. 36, Nanjing 210009, People’s Republic of China

## Abstract

The asymmetric unit of the title compound, C_16_H_17_FN_2_O_4_S, contains three independent mol­ecules, in which the pyrimidine and benzene rings are oriented at dihedral angles of 41.72 (3)°, 26.21 (3)° and 36.49 (3)°. Intra­molecular C—H⋯O hydrogen bonds result in the formation of two six- and one seven-membered non-planar rings, which have have twist conformations. In the crystal structure, inter­molecular C—H⋯O hydrogen bonds link the mol­ecules.

## Related literature

For related literature, see: Gompper *et al.* (1997 ▶); Laufer & Wagner (2002 ▶). For bond-length data, see: Allen *et al.* (1987 ▶).
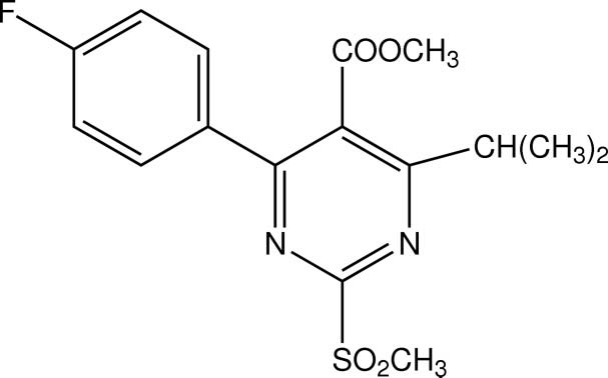


## Experimental

### 

#### Crystal data

C_16_H_17_FN_2_O_4_S
*M* *_r_* = 352.39
Monoclinic, 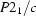

*a* = 28.875 (6) Å
*b* = 9.887 (2) Å
*c* = 18.400 (4) Å
β = 98.09 (3)°
*V* = 5200.7 (18) Å^3^
*Z* = 12
Mo *K*α radiation
μ = 0.22 mm^−1^
*T* = 294 (2) K
0.40 × 0.20 × 0.20 mm

#### Data collection

Enraf–Nonius CAD-4 diffractometer
Absorption correction: ψ scan (North *et al.*, 1968 ▶) *T*_min_ = 0.918, *T*_max_ = 0.958
10466 measured reflections
10124 independent reflections
4777 reflections with *I* > 2σ(*I*)
*R*_int_ = 0.066
3 standard reflections frequency: 120 min intensity decay: none

#### Refinement

*R*[*F* ^2^ > 2σ(*F* ^2^)] = 0.079
*wR*(*F* ^2^) = 0.206
*S* = 1.03
10124 reflections
637 parameters
H-atom parameters constrained
Δρ_max_ = 0.42 e Å^−3^
Δρ_min_ = −0.58 e Å^−3^

### 

Data collection: *CAD-4 Software* (Enraf–Nonius, 1989 ▶); cell refinement: *CAD-4 Software*; data reduction: *XCAD4* (Harms & Wocadlo, 1995 ▶); program(s) used to solve structure: *SHELXS97* (Sheldrick, 2008 ▶); program(s) used to refine structure: *SHELXL97* (Sheldrick, 2008 ▶); molecular graphics: *PLATON* (Spek, 2003 ▶); software used to prepare material for publication: *SHELXTL* (Sheldrick, 2008 ▶).

## Supplementary Material

Crystal structure: contains datablocks D, I. DOI: 10.1107/S1600536808013536/hk2457sup1.cif

Structure factors: contains datablocks I. DOI: 10.1107/S1600536808013536/hk2457Isup2.hkl

Additional supplementary materials:  crystallographic information; 3D view; checkCIF report

## Figures and Tables

**Table 1:** Hydrogen-bond geometry (Å, °)

*D*—H⋯*A*	*D*—H	H⋯*A*	*D*⋯*A*	*D*—H⋯*A*
C3—H3*B*⋯O1	0.98	2.54	3.089 (7)	115
C17—H17*C*⋯F1	0.96	2.51	3.191 (8)	128
C19—H19*A*⋯O6	0.98	2.55	3.224 (7)	126
C28—H28*A*⋯O6	0.93	2.56	3.378 (6)	147
C8—H8*B*⋯O3^i^	0.96	2.48	3.373 (6)	155
C35—H35*A*⋯O12^ii^	0.98	2.57	3.484 (6)	154
C42—H42*A*⋯O9^iii^	0.96	2.43	3.229 (6)	141
C48—H48*A*⋯O12^iv^	0.93	2.42	3.224 (6)	146

Symmetry codes: (i) 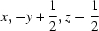; (ii) 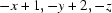; (iii) 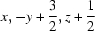; (iv) 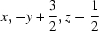.

**Fig. 1.**
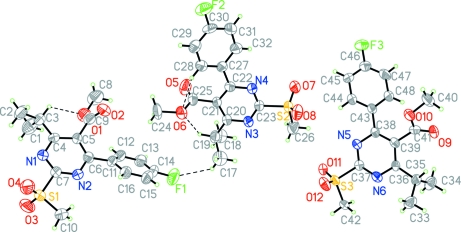
The molecular structure of the title molecule, with the atom-numbering scheme. Displacement ellipsoids are drawn at the 30% probability level. Hydrogen bonds are shown as dashed lines.

**Fig. 2.**
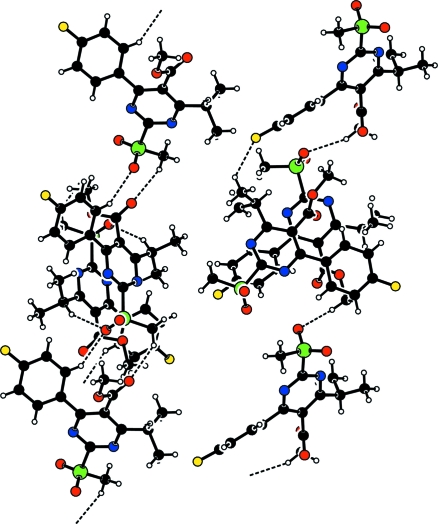
A partial packing diagram of (I). Hydrogen bonds are shown as dashed lines.

## supplementary crystallographic information

### Comment

Some derivatives of pyrimidine are important chemical materials. We report herein the crystal structure of the title compound, (I).

The asymmetric unit of the title compound, (I), (Fig. 1) contains three independent molecules. Rings A (N1/N2/C4-C7), B (C11-C16), C (N3/N4/C20-C23), D (C27-C32), E (N5/N6/C36-C9) and F (C43-C48) are, of course, planar, and the dihedral angles between them are A/B = 41.72 (3)°, B/C = 26.21 (3)° and D/E = 36.49 (3)°. The intramolecular C-H···O hydrogen bonds (Table 1) result in the formation of two six- and one seven-membered non-planar rings: G (O1/C3-C5/C9/H3B), H (O6/C19-C21/C25/H19A) and I (O6/C21/C22/C25/C27/C28/H28A). They adopt twisted conformations, having total puckering amplitudes, Q_T_, of 0.788 (3) Å, 1.806 (3) Å and 1.290 (3) Å, respectively.

In the crystal structure, intermolecular C-H···O hydrogen bonds (Table 1) link the molecules (Fig. 2), in which they may be effective in the stabilization of the structure.

### Experimental

For the preparation of the title compound, methyl4-(4-fluorophenyl)-6-isopropyl -2-(methylthio)pyrimidine-5-carboxylate (100 g, 312.50 mmol), ammonium molybdate tetrahydrate (4.95 g, 4.00 mmol) and sulfuric acid (0.32 g, 3.26 mmol) were added to methanol (1000 ml) in a round bottom flask, and then stirred for 1 h at 303 K. H_2_O_2_ (106.2 ml) was added dropwise in 30 min, and stirred for 2 h. The mixture was stirred for 5 h, by increasing the temperatue to 323 K. After completion of the reaction, the mixture was cooled to 273 K and stirred for 1 h. It was filtered, washed with water, and then dried (yield; 88%). Crystals of (I) suitable for X-ray analysis were obtained by slow evaporation of an ethanol solution.

### Refinement

H atoms were positioned geometrically, with C-H = 0.93, 0.98 and 0.96 Å for aromatic, methine and methyl H, respectively, and constrained to ride on their parent atoms with U_iso_(H) = xU_eq_(C), where x = 1.5 for methyl H, and x = 1.2 for all other H atoms.

### Crystal data

**Table:**

C_16_H_17_FN_2_O_4_S	*F*_000_ = 2208
*M**_r_* = 352.39	*D*_x_ = 1.350 Mg m^−^^3^
Monoclinic, *P*2_1_/*c*	Mo *K*α radiation λ = 0.71073 Å
Hall symbol: -P 2ybc	Cell parameters from 25 reflections
*a* = 28.875 (6) Å	θ = 9–12º
*b* = 9.887 (2) Å	µ = 0.22 mm^−^^1^
*c* = 18.400 (4) Å	*T* = 294 (2) K
β = 98.09 (3)º	Block, colorless
*V* = 5200.7 (18) Å^3^	0.40 × 0.20 × 0.20 mm
*Z* = 12	

### Data collection

**Table:**

Enraf–Nonius CAD-4 diffractometer	*R*_int_ = 0.066
Radiation source: fine-focus sealed tube	θ_max_ = 26.0º
Monochromator: graphite	θ_min_ = 1.4º
*T* = 294(2) K	*h* = −34→34
ω/2θ scans	*k* = 0→11
Absorption correction: ψ scan(North *et al.*, 1968)	*l* = 0→22
*T*_min_ = 0.918, *T*_max_ = 0.958	3 standard reflections
10466 measured reflections	every 120 min
10124 independent reflections	intensity decay: none
4777 reflections with *I* > 2σ(*I*)	

### Refinement

**Table:**

Refinement on *F*^2^	Secondary atom site location: difference Fourier map
Least-squares matrix: full	Hydrogen site location: inferred from neighbouring sites
*R*[*F*^2^ > 2σ(*F*^2^)] = 0.079	H-atom parameters constrained
*wR*(*F*^2^) = 0.206	*w* = 1/[σ^2^(*F*_o_^2^) + (0.080*P*)^2^] where *P* = (*F*_o_^2^ + 2*F*_c_^2^)/3
*S* = 1.03	(Δ/σ)_max_ < 0.001
10124 reflections	Δρ_max_ = 0.42 e Å^−^^3^
637 parameters	Δρ_min_ = −0.58 e Å^−^^3^
Primary atom site location: structure-invariant direct methods	Extinction correction: none

### Special details

**Table:**

Geometry. All e.s.d.'s (except the e.s.d. in the dihedral angle between two l.s. planes) are estimated using the full covariance matrix. The cell e.s.d.'s are taken into account individually in the estimation of e.s.d.'s in distances, angles and torsion angles; correlations between e.s.d.'s in cell parameters are only used when they are defined by crystal symmetry. An approximate (isotropic) treatment of cell e.s.d.'s is used for estimating e.s.d.'s involving l.s. planes.
Refinement. Refinement of F^2^ against ALL reflections. The weighted R-factor wR and goodness of fit S are based on F^2^, conventional R-factors R are based on F, with F set to zero for negative F^2^. The threshold expression of F^2^ > 2sigma(F^2^) is used only for calculating R-factors(gt) etc. and is not relevant to the choice of reflections for refinement. R-factors based on F^2^ are statistically about twice as large as those based on F, and R- factors based on ALL data will be even larger.

### Fractional atomic coordinates and isotropic or equivalent isotropic displacement parameters (Å^2^)

**Table:**

	*x*	*y*	*z*	*U*_iso_*/*U*_eq_	
S1	0.14585 (5)	0.53855 (18)	0.81769 (7)	0.0619 (4)	
O1	0.06944 (14)	0.1784 (4)	0.5211 (2)	0.0781 (12)	
O2	0.08271 (14)	0.3803 (5)	0.4713 (2)	0.0777 (13)	
O3	0.15061 (15)	0.4344 (5)	0.8708 (2)	0.0881 (14)	
O4	0.11470 (14)	0.6475 (4)	0.8258 (2)	0.0848 (13)	
N1	0.08452 (15)	0.4164 (4)	0.7203 (2)	0.0531 (11)	
N2	0.15938 (14)	0.4561 (5)	0.6845 (2)	0.0538 (11)	
F1	0.29201 (13)	0.3184 (4)	0.4561 (2)	0.1078 (14)	
C1	0.0211 (2)	0.1644 (6)	0.6804 (4)	0.096 (2)	
H1B	0.0424	0.1062	0.6596	0.144*	
H1C	−0.0097	0.1262	0.6722	0.144*	
H1D	0.0310	0.1735	0.7322	0.144*	
C2	−0.0138 (2)	0.3993 (7)	0.6734 (4)	0.084 (2)	
H2B	−0.0142	0.4842	0.6479	0.125*	
H2C	−0.0043	0.4138	0.7249	0.125*	
H2D	−0.0445	0.3602	0.6657	0.125*	
C3	0.02057 (18)	0.3038 (5)	0.6440 (3)	0.0578 (14)	
H3B	0.0105	0.2918	0.5913	0.069*	
C4	0.06945 (16)	0.3576 (5)	0.6538 (3)	0.0466 (12)	
C5	0.09929 (17)	0.3535 (5)	0.6005 (3)	0.0455 (12)	
C6	0.14539 (18)	0.3964 (5)	0.6189 (3)	0.0463 (12)	
C7	0.12823 (17)	0.4613 (5)	0.7291 (3)	0.0484 (13)	
C8	0.0534 (2)	0.1242 (8)	0.4483 (4)	0.109 (3)	
H8A	0.0455	0.0305	0.4523	0.164*	
H8B	0.0779	0.1330	0.4182	0.164*	
H8C	0.0264	0.1734	0.4265	0.164*	
C9	0.08202 (18)	0.3085 (6)	0.5234 (3)	0.0559 (14)	
C10	0.20124 (19)	0.6025 (7)	0.8115 (3)	0.0742 (18)	
H10A	0.2133	0.6466	0.8567	0.111*	
H10B	0.1993	0.6664	0.7719	0.111*	
H10C	0.2217	0.5297	0.8025	0.111*	
C11	0.18219 (17)	0.3796 (5)	0.5712 (3)	0.0474 (13)	
C12	0.18688 (19)	0.2589 (6)	0.5343 (3)	0.0569 (14)	
H12A	0.1652	0.1901	0.5365	0.068*	
C13	0.22339 (19)	0.2399 (6)	0.4946 (3)	0.0616 (15)	
H13A	0.2263	0.1598	0.4691	0.074*	
C14	0.2548 (2)	0.3406 (7)	0.4936 (3)	0.0682 (17)	
C15	0.2519 (2)	0.4624 (7)	0.5281 (3)	0.0737 (18)	
H15A	0.2736	0.5306	0.5245	0.088*	
C16	0.21536 (19)	0.4801 (6)	0.5686 (3)	0.0577 (14)	
H16A	0.2131	0.5602	0.5943	0.069*	
S2	0.30615 (5)	0.60184 (15)	0.02405 (7)	0.0538 (4)	
O5	0.14659 (15)	0.3704 (4)	0.2138 (2)	0.0810 (13)	
O6	0.18226 (13)	0.5146 (4)	0.29765 (19)	0.0654 (11)	
O7	0.28275 (14)	0.5614 (5)	−0.04534 (19)	0.0805 (13)	
O8	0.32002 (14)	0.7407 (4)	0.0338 (2)	0.0748 (12)	
N3	0.28601 (14)	0.4816 (4)	0.1474 (2)	0.0481 (10)	
N4	0.22682 (15)	0.6175 (4)	0.0759 (2)	0.0514 (11)	
F2	0.02822 (11)	0.8653 (4)	0.0472 (2)	0.0861 (11)	
C17	0.3160 (2)	0.4249 (7)	0.3020 (4)	0.088	
H17A	0.3074	0.5104	0.3210	0.132*	
H17B	0.3400	0.4383	0.2716	0.132*	
H17C	0.3274	0.3660	0.3420	0.132*	
C18	0.2865 (2)	0.2228 (6)	0.2288 (4)	0.086	
H18A	0.2589	0.1828	0.2020	0.128*	
H18B	0.2982	0.1656	0.2694	0.128*	
H18C	0.3099	0.2329	0.1970	0.128*	
C19	0.27420 (19)	0.3627 (6)	0.2577 (3)	0.0592 (15)	
H19A	0.2498	0.3512	0.2892	0.071*	
C20	0.25592 (17)	0.4539 (5)	0.1949 (3)	0.0474 (12)	
C21	0.20956 (17)	0.5053 (5)	0.1837 (3)	0.0440 (12)	
C22	0.19646 (17)	0.5897 (5)	0.1238 (3)	0.0446 (12)	
C23	0.26833 (18)	0.5614 (5)	0.0913 (3)	0.0515 (13)	
C24	0.1506 (2)	0.4707 (7)	0.3498 (3)	0.094 (2)	
H24A	0.1581	0.5191	0.3952	0.141*	
H24B	0.1544	0.3754	0.3588	0.141*	
H24C	0.1188	0.4891	0.3293	0.141*	
C25	0.1753 (2)	0.4533 (6)	0.2322 (3)	0.0517 (13)	
C26	0.3550 (2)	0.4989 (7)	0.0467 (4)	0.088 (2)	
H26A	0.3768	0.5148	0.0128	0.132*	
H26B	0.3455	0.4057	0.0440	0.132*	
H26C	0.3695	0.5194	0.0956	0.132*	
C27	0.15002 (17)	0.6563 (5)	0.1054 (3)	0.0460 (12)	
C28	0.12203 (18)	0.6904 (5)	0.1578 (3)	0.0545 (14)	
H28A	0.1313	0.6676	0.2067	0.065*	
C29	0.08071 (19)	0.7578 (6)	0.1378 (3)	0.0629 (16)	
H29A	0.0614	0.7781	0.1727	0.075*	
C30	0.06822 (19)	0.7948 (6)	0.0661 (3)	0.0653 (16)	
C31	0.0941 (2)	0.7609 (7)	0.0118 (3)	0.0755 (19)	
H31A	0.0842	0.7836	−0.0370	0.091*	
C32	0.13540 (19)	0.6920 (6)	0.0324 (3)	0.0663 (16)	
H32A	0.1539	0.6687	−0.0032	0.080*	
S3	0.49545 (4)	0.64141 (12)	0.08480 (6)	0.0412 (3)	
O9	0.51612 (12)	0.9595 (4)	−0.23235 (17)	0.0565 (9)	
O10	0.44927 (12)	1.0270 (3)	−0.19608 (17)	0.0496 (9)	
O11	0.47036 (11)	0.5155 (3)	0.07353 (16)	0.0494 (9)	
O12	0.47873 (13)	0.7416 (3)	0.13142 (17)	0.0572 (10)	
N5	0.45677 (13)	0.7060 (4)	−0.04859 (19)	0.0377 (9)	
N6	0.53247 (14)	0.7946 (4)	−0.0098 (2)	0.0429 (10)	
F3	0.29254 (12)	0.6619 (4)	−0.30536 (18)	0.1047 (14)	
C33	0.59905 (19)	0.9978 (6)	−0.0043 (3)	0.0654 (16)	
H33A	0.5771	1.0440	0.0214	0.098*	
H33B	0.6241	1.0580	−0.0112	0.098*	
H33C	0.6114	0.9208	0.0238	0.098*	
C34	0.60853 (18)	0.8738 (5)	−0.1197 (3)	0.0620 (15)	
H34A	0.5927	0.8462	−0.1667	0.093*	
H34B	0.6198	0.7954	−0.0919	0.093*	
H34C	0.6344	0.9313	−0.1264	0.093*	
C35	0.57467 (17)	0.9510 (5)	−0.0785 (3)	0.0437 (12)	
H35A	0.5648	1.0320	−0.1073	0.052*	
C36	0.53143 (15)	0.8689 (4)	−0.0722 (2)	0.0335 (10)	
C37	0.49502 (16)	0.7209 (4)	−0.0025 (2)	0.0320 (10)	
C38	0.45494 (16)	0.7761 (4)	−0.1125 (2)	0.0346 (10)	
C39	0.49147 (16)	0.8610 (4)	−0.1245 (2)	0.0343 (10)	
C40	0.4413 (2)	1.1136 (5)	−0.2599 (3)	0.0640 (16)	
H40A	0.4129	1.1637	−0.2592	0.096*	
H40B	0.4387	1.0593	−0.3036	0.096*	
H40C	0.4670	1.1753	−0.2593	0.096*	
C41	0.48822 (16)	0.9515 (5)	−0.1902 (3)	0.0397 (11)	
C42	0.55448 (16)	0.6089 (5)	0.1152 (2)	0.0459 (12)	
H42A	0.5576	0.5657	0.1623	0.069*	
H42B	0.5715	0.6926	0.1192	0.069*	
H42C	0.5668	0.5507	0.0808	0.069*	
C43	0.41179 (16)	0.7494 (4)	−0.1652 (2)	0.0372 (11)	
C44	0.37025 (18)	0.7269 (5)	−0.1387 (3)	0.0524 (13)	
H44A	0.3693	0.7319	−0.0885	0.063*	
C45	0.33026 (18)	0.6972 (6)	−0.1854 (3)	0.0682 (17)	
H45A	0.3022	0.6821	−0.1674	0.082*	
C46	0.3326 (2)	0.6902 (6)	−0.2588 (3)	0.0651 (16)	
C47	0.3727 (2)	0.7107 (6)	−0.2877 (3)	0.0601 (15)	
H47A	0.3730	0.7049	−0.3380	0.072*	
C48	0.41319 (18)	0.7402 (5)	−0.2408 (2)	0.0467 (12)	
H48A	0.4411	0.7539	−0.2594	0.056*	

### Atomic displacement parameters (Å^2^)

**Table:**

	*U*^11^	*U*^22^	*U*^33^	*U*^12^	*U*^13^	*U*^23^
S1	0.0506 (9)	0.0947 (11)	0.0395 (8)	−0.0079 (9)	0.0030 (6)	−0.0031 (8)
O1	0.070 (3)	0.091 (3)	0.073 (3)	−0.008 (2)	0.011 (2)	−0.030 (2)
O2	0.076 (3)	0.106 (3)	0.049 (2)	0.006 (2)	0.001 (2)	0.003 (2)
O3	0.095 (3)	0.112 (3)	0.052 (2)	−0.013 (3)	−0.004 (2)	0.020 (3)
O4	0.066 (3)	0.111 (3)	0.079 (3)	0.016 (3)	0.014 (2)	−0.035 (3)
N1	0.043 (3)	0.073 (3)	0.042 (2)	0.002 (2)	0.005 (2)	0.002 (2)
N2	0.040 (2)	0.075 (3)	0.046 (3)	−0.002 (2)	0.004 (2)	0.000 (2)
F1	0.071 (3)	0.155 (4)	0.108 (3)	−0.005 (2)	0.047 (2)	−0.024 (3)
C1	0.074 (5)	0.082 (5)	0.134 (7)	−0.021 (4)	0.020 (4)	0.014 (5)
C2	0.047 (4)	0.109 (5)	0.097 (5)	−0.007 (4)	0.015 (3)	−0.023 (4)
C3	0.044 (3)	0.072 (4)	0.058 (3)	−0.001 (3)	0.009 (3)	−0.005 (3)
C4	0.035 (3)	0.056 (3)	0.046 (3)	−0.005 (2)	−0.002 (2)	−0.003 (3)
C5	0.040 (3)	0.055 (3)	0.043 (3)	0.004 (2)	0.010 (2)	0.000 (3)
C6	0.049 (3)	0.044 (3)	0.046 (3)	−0.005 (2)	0.008 (2)	0.004 (2)
C7	0.039 (3)	0.061 (3)	0.045 (3)	0.000 (3)	0.006 (2)	0.002 (3)
C8	0.079 (5)	0.137 (7)	0.111 (6)	−0.019 (5)	0.009 (4)	−0.071 (5)
C9	0.041 (3)	0.063 (4)	0.063 (4)	0.003 (3)	0.007 (3)	−0.013 (3)
C10	0.050 (4)	0.112 (5)	0.059 (4)	−0.014 (4)	0.001 (3)	−0.008 (4)
C11	0.037 (3)	0.058 (3)	0.049 (3)	0.004 (3)	0.010 (2)	0.003 (3)
C12	0.044 (3)	0.066 (4)	0.061 (4)	0.004 (3)	0.009 (3)	0.005 (3)
C13	0.054 (4)	0.068 (4)	0.063 (4)	0.013 (3)	0.009 (3)	−0.008 (3)
C14	0.045 (3)	0.102 (5)	0.062 (4)	−0.008 (3)	0.021 (3)	−0.006 (4)
C15	0.055 (4)	0.096 (5)	0.073 (4)	−0.026 (4)	0.018 (3)	−0.006 (4)
C16	0.054 (4)	0.062 (3)	0.058 (4)	−0.006 (3)	0.012 (3)	−0.003 (3)
S2	0.0423 (8)	0.0774 (10)	0.0432 (8)	−0.0012 (7)	0.0115 (6)	0.0141 (7)
O5	0.074 (3)	0.084 (3)	0.092 (3)	−0.028 (3)	0.034 (2)	−0.005 (3)
O6	0.063 (3)	0.089 (3)	0.047 (2)	0.007 (2)	0.0173 (19)	−0.002 (2)
O7	0.066 (3)	0.135 (4)	0.042 (2)	−0.025 (3)	0.0103 (19)	0.003 (2)
O8	0.074 (3)	0.073 (3)	0.082 (3)	−0.016 (2)	0.029 (2)	0.013 (2)
N3	0.048 (3)	0.056 (3)	0.042 (2)	−0.001 (2)	0.009 (2)	0.014 (2)
N4	0.042 (3)	0.070 (3)	0.042 (2)	0.000 (2)	0.009 (2)	0.011 (2)
F2	0.049 (2)	0.103 (3)	0.103 (3)	0.0164 (19)	0.0001 (18)	0.012 (2)
C17	0.088	0.088	0.088	0.000	0.012	0.000
C18	0.086	0.086	0.086	0.000	0.012	0.000
C19	0.050 (3)	0.074 (4)	0.057 (3)	0.005 (3)	0.021 (3)	0.010 (3)
C20	0.045 (3)	0.056 (3)	0.041 (3)	−0.004 (3)	0.009 (2)	0.006 (3)
C21	0.043 (3)	0.055 (3)	0.035 (3)	−0.008 (2)	0.009 (2)	0.000 (2)
C22	0.043 (3)	0.049 (3)	0.041 (3)	−0.003 (2)	0.003 (2)	−0.001 (2)
C23	0.044 (3)	0.072 (4)	0.040 (3)	−0.007 (3)	0.009 (2)	0.002 (3)
C24	0.087 (5)	0.145 (6)	0.061 (4)	0.019 (5)	0.045 (4)	0.030 (4)
C25	0.056 (4)	0.053 (3)	0.050 (3)	0.005 (3)	0.020 (3)	0.008 (3)
C26	0.057 (4)	0.119 (6)	0.094 (5)	0.026 (4)	0.033 (4)	0.032 (4)
C27	0.038 (3)	0.055 (3)	0.045 (3)	0.007 (2)	0.007 (2)	−0.001 (3)
C28	0.051 (3)	0.064 (4)	0.047 (3)	0.003 (3)	0.003 (3)	0.000 (3)
C29	0.049 (4)	0.075 (4)	0.067 (4)	0.011 (3)	0.016 (3)	−0.003 (3)
C30	0.033 (3)	0.083 (4)	0.076 (4)	0.013 (3)	−0.008 (3)	0.008 (4)
C31	0.055 (4)	0.113 (5)	0.059 (4)	0.022 (4)	0.009 (3)	0.025 (4)
C32	0.047 (3)	0.099 (5)	0.052 (4)	0.012 (3)	0.005 (3)	0.002 (3)
S3	0.0468 (7)	0.0510 (7)	0.0270 (6)	0.0044 (6)	0.0087 (5)	0.0025 (6)
O9	0.057 (2)	0.079 (2)	0.038 (2)	−0.002 (2)	0.0198 (18)	0.0074 (19)
O10	0.057 (2)	0.054 (2)	0.0382 (19)	0.0029 (18)	0.0064 (16)	0.0090 (17)
O11	0.050 (2)	0.060 (2)	0.041 (2)	−0.0063 (17)	0.0139 (16)	0.0081 (17)
O12	0.076 (3)	0.061 (2)	0.0372 (19)	0.015 (2)	0.0180 (18)	0.0050 (18)
N5	0.043 (2)	0.040 (2)	0.030 (2)	0.0006 (18)	0.0060 (17)	0.0033 (17)
N6	0.053 (2)	0.041 (2)	0.035 (2)	0.0003 (19)	0.0030 (18)	0.0041 (18)
F3	0.067 (2)	0.172 (4)	0.066 (2)	−0.050 (3)	−0.0211 (19)	0.012 (2)
C33	0.056 (4)	0.080 (4)	0.061 (4)	−0.024 (3)	0.010 (3)	−0.023 (3)
C34	0.054 (3)	0.068 (4)	0.069 (4)	−0.007 (3)	0.026 (3)	−0.017 (3)
C35	0.050 (3)	0.041 (3)	0.041 (3)	−0.013 (2)	0.007 (2)	−0.003 (2)
C36	0.039 (2)	0.032 (2)	0.032 (2)	−0.001 (2)	0.0138 (19)	−0.002 (2)
C37	0.039 (3)	0.022 (2)	0.037 (2)	0.0067 (19)	0.011 (2)	−0.0030 (19)
C38	0.041 (3)	0.035 (2)	0.030 (2)	0.003 (2)	0.011 (2)	−0.002 (2)
C39	0.044 (3)	0.031 (2)	0.028 (2)	0.006 (2)	0.0080 (19)	−0.0020 (19)
C40	0.082 (4)	0.060 (4)	0.047 (3)	0.001 (3)	0.002 (3)	0.017 (3)
C41	0.035 (3)	0.044 (3)	0.038 (3)	0.003 (2)	−0.003 (2)	−0.004 (2)
C42	0.044 (3)	0.060 (3)	0.032 (3)	−0.001 (3)	0.001 (2)	0.010 (2)
C43	0.044 (3)	0.036 (3)	0.032 (2)	−0.003 (2)	0.006 (2)	0.004 (2)
C44	0.050 (3)	0.073 (4)	0.034 (3)	−0.008 (3)	0.010 (2)	0.005 (3)
C45	0.036 (3)	0.119 (5)	0.048 (3)	−0.019 (3)	−0.002 (3)	0.011 (3)
C46	0.053 (4)	0.087 (4)	0.051 (4)	−0.022 (3)	−0.006 (3)	0.006 (3)
C47	0.070 (4)	0.074 (4)	0.033 (3)	−0.008 (3)	−0.001 (3)	0.000 (3)
C48	0.049 (3)	0.059 (3)	0.033 (3)	−0.003 (3)	0.009 (2)	−0.001 (2)

### Geometric parameters (Å, °)

**Table:**

S1—O3	1.412 (4)	C19—H19A	0.9800
S1—O4	1.425 (4)	C20—C21	1.420 (7)
S1—C7	1.807 (5)	C21—C22	1.391 (6)
S1—C10	1.738 (6)	C21—C25	1.513 (7)
O1—C8	1.456 (7)	C22—C27	1.490 (6)
O1—C9	1.335 (6)	C24—H24A	0.9600
O2—C9	1.195 (6)	C24—H24B	0.9600
N1—C4	1.369 (6)	C24—H24C	0.9600
N1—C7	1.326 (6)	C26—H26A	0.9600
N2—C6	1.353 (6)	C26—H26B	0.9600
N2—C7	1.301 (6)	C26—H26C	0.9600
F1—C14	1.374 (6)	C27—C28	1.384 (6)
C1—C3	1.530 (8)	C27—C32	1.395 (7)
C1—H1B	0.9600	C28—C29	1.371 (7)
C1—H1C	0.9600	C28—H28A	0.9300
C1—H1D	0.9600	C29—C30	1.367 (7)
C2—C3	1.523 (7)	C29—H29A	0.9300
C2—H2B	0.9600	C30—C31	1.371 (8)
C2—H2C	0.9600	C31—C32	1.380 (7)
C2—H2D	0.9600	C31—H31A	0.9300
C3—C4	1.495 (7)	C32—H32A	0.9300
C3—H3B	0.9800	S3—O11	1.440 (3)
C4—C5	1.395 (6)	S3—O12	1.438 (3)
C5—C6	1.393 (6)	S3—C37	1.786 (4)
C5—C9	1.503 (7)	S3—C42	1.747 (5)
C6—C11	1.480 (6)	O9—C41	1.198 (5)
C8—H8A	0.9600	O10—C40	1.446 (5)
C8—H8B	0.9600	O10—C41	1.341 (5)
C8—H8C	0.9600	N5—C37	1.303 (5)
C10—H10A	0.9600	N5—C38	1.360 (5)
C10—H10B	0.9600	N6—C36	1.360 (5)
C10—H10C	0.9600	N6—C37	1.326 (5)
C11—C12	1.388 (7)	F3—C46	1.369 (6)
C11—C16	1.386 (7)	C33—C35	1.517 (6)
C12—C13	1.378 (7)	C33—H33A	0.9600
C12—H12A	0.9300	C33—H33B	0.9600
C13—C14	1.349 (8)	C33—H33C	0.9600
C13—H13A	0.9300	C34—C35	1.523 (6)
C14—C15	1.369 (8)	C34—H34A	0.9600
C15—C16	1.385 (7)	C34—H34B	0.9600
C15—H15A	0.9300	C34—H34C	0.9600
C16—H16A	0.9300	C35—C36	1.508 (6)
S2—O7	1.416 (4)	C35—H35A	0.9800
S2—O8	1.435 (4)	C36—C39	1.397 (6)
S2—C23	1.807 (5)	C38—C39	1.390 (6)
S2—C26	1.742 (6)	C38—C43	1.491 (6)
O5—C25	1.180 (6)	C39—C41	1.496 (6)
O6—C24	1.481 (6)	C40—H40A	0.9600
O6—C25	1.338 (6)	C40—H40B	0.9600
N3—C20	1.346 (6)	C40—H40C	0.9600
N3—C23	1.341 (6)	C42—H42A	0.9600
N4—C22	1.356 (6)	C42—H42B	0.9600
N4—C23	1.315 (6)	C42—H42C	0.9600
F2—C30	1.352 (6)	C43—C44	1.375 (6)
C17—C19	1.490 (8)	C43—C48	1.401 (6)
C17—H17A	0.9600	C44—C45	1.370 (7)
C17—H17B	0.9600	C44—H44A	0.9300
C17—H17C	0.9600	C45—C46	1.363 (7)
C18—C19	1.541 (8)	C45—H45A	0.9300
C18—H18A	0.9600	C46—C47	1.355 (7)
C18—H18B	0.9600	C47—C48	1.382 (7)
C18—H18C	0.9600	C47—H47A	0.9300
C19—C20	1.501 (7)	C48—H48A	0.9300
			
O3—S1—O4	118.7 (3)	N4—C23—N3	130.6 (4)
O3—S1—C7	107.6 (3)	N4—C23—S2	112.3 (4)
O3—S1—C10	108.0 (3)	N3—C23—S2	117.1 (4)
O4—S1—C7	108.2 (2)	O6—C24—H24A	109.5
O4—S1—C10	109.4 (3)	O6—C24—H24B	109.5
C10—S1—C7	103.9 (3)	H24A—C24—H24B	109.5
C9—O1—C8	115.7 (5)	O6—C24—H24C	109.5
C7—N1—C4	115.4 (4)	H24A—C24—H24C	109.5
C7—N2—C6	115.7 (4)	H24B—C24—H24C	109.5
C3—C1—H1B	109.5	O5—C25—O6	125.7 (5)
C3—C1—H1C	109.5	O5—C25—C21	124.0 (5)
H1B—C1—H1C	109.5	O6—C25—C21	110.4 (5)
C3—C1—H1D	109.5	S2—C26—H26A	109.5
H1B—C1—H1D	109.5	S2—C26—H26B	109.5
H1C—C1—H1D	109.5	H26A—C26—H26B	109.5
C3—C2—H2B	109.5	S2—C26—H26C	109.5
C3—C2—H2C	109.5	H26A—C26—H26C	109.5
H2B—C2—H2C	109.5	H26B—C26—H26C	109.5
C3—C2—H2D	109.5	C28—C27—C32	118.8 (5)
H2B—C2—H2D	109.5	C28—C27—C22	123.0 (5)
H2C—C2—H2D	109.5	C32—C27—C22	118.1 (4)
C4—C3—C2	112.8 (5)	C29—C28—C27	120.2 (5)
C4—C3—C1	108.4 (5)	C29—C28—H28A	119.9
C2—C3—C1	111.8 (5)	C27—C28—H28A	119.9
C4—C3—H3B	107.9	C30—C29—C28	119.3 (5)
C2—C3—H3B	107.9	C30—C29—H29A	120.3
C1—C3—H3B	107.9	C28—C29—H29A	120.3
N1—C4—C5	119.4 (4)	F2—C30—C29	119.1 (5)
N1—C4—C3	115.8 (4)	F2—C30—C31	118.1 (5)
C5—C4—C3	124.8 (5)	C29—C30—C31	122.8 (5)
C6—C5—C4	118.9 (5)	C30—C31—C32	117.3 (6)
C6—C5—C9	119.9 (4)	C30—C31—H31A	121.3
C4—C5—C9	121.1 (5)	C32—C31—H31A	121.3
N2—C6—C5	120.4 (4)	C31—C32—C27	121.5 (5)
N2—C6—C11	115.4 (4)	C31—C32—H32A	119.3
C5—C6—C11	124.2 (5)	C27—C32—H32A	119.3
N2—C7—N1	129.8 (5)	O11—S3—C37	108.1 (2)
N2—C7—S1	117.0 (4)	O11—S3—C42	109.6 (2)
N1—C7—S1	113.2 (4)	O12—S3—O11	118.5 (2)
O1—C8—H8A	109.5	O12—S3—C37	105.83 (19)
O1—C8—H8B	109.5	O12—S3—C42	109.3 (2)
H8A—C8—H8B	109.5	C42—S3—C37	104.6 (2)
O1—C8—H8C	109.5	C41—O10—C40	115.5 (4)
H8A—C8—H8C	109.5	C37—N5—C38	115.6 (4)
H8B—C8—H8C	109.5	C37—N6—C36	117.4 (4)
O2—C9—O1	125.6 (6)	C35—C33—H33A	109.5
O2—C9—C5	122.7 (5)	C35—C33—H33B	109.5
O1—C9—C5	111.6 (5)	H33A—C33—H33B	109.5
S1—C10—H10A	109.5	C35—C33—H33C	109.5
S1—C10—H10B	109.5	H33A—C33—H33C	109.5
H10A—C10—H10B	109.5	H33B—C33—H33C	109.5
S1—C10—H10C	109.5	C35—C34—H34A	109.5
H10A—C10—H10C	109.5	C35—C34—H34B	109.5
H10B—C10—H10C	109.5	H34A—C34—H34B	109.5
C16—C11—C12	118.9 (5)	C35—C34—H34C	109.5
C16—C11—C6	119.8 (5)	H34A—C34—H34C	109.5
C12—C11—C6	120.9 (5)	H34B—C34—H34C	109.5
C13—C12—C11	120.7 (5)	C36—C35—C33	112.3 (4)
C13—C12—H12A	119.6	C36—C35—C34	111.4 (4)
C11—C12—H12A	119.6	C33—C35—C34	110.6 (4)
C14—C13—C12	118.2 (5)	C36—C35—H35A	107.4
C14—C13—H13A	120.9	C33—C35—H35A	107.4
C12—C13—H13A	120.9	C34—C35—H35A	107.4
C13—C14—C15	123.9 (5)	N6—C36—C39	118.0 (4)
C13—C14—F1	117.6 (6)	N6—C36—C35	115.9 (4)
C15—C14—F1	118.5 (6)	C39—C36—C35	126.1 (4)
C14—C15—C16	117.5 (5)	N5—C37—N6	128.6 (4)
C14—C15—H15A	121.3	N5—C37—S3	115.7 (3)
C16—C15—H15A	121.3	N6—C37—S3	115.5 (3)
C15—C16—C11	120.7 (5)	N5—C38—C39	120.5 (4)
C15—C16—H16A	119.7	N5—C38—C43	113.5 (4)
C11—C16—H16A	119.7	C39—C38—C43	126.0 (4)
O7—S2—O8	118.2 (3)	C38—C39—C36	119.7 (4)
O7—S2—C23	107.3 (2)	C38—C39—C41	121.8 (4)
O7—S2—C26	108.5 (3)	C36—C39—C41	118.4 (4)
O8—S2—C23	108.1 (2)	O10—C40—H40A	109.5
O8—S2—C26	109.1 (3)	O10—C40—H40B	109.5
C26—S2—C23	104.7 (3)	H40A—C40—H40B	109.5
C25—O6—C24	114.9 (5)	O10—C40—H40C	109.5
C23—N3—C20	114.2 (4)	H40A—C40—H40C	109.5
C23—N4—C22	115.3 (4)	H40B—C40—H40C	109.5
C19—C17—H17A	109.5	O9—C41—O10	122.9 (4)
C19—C17—H17B	109.5	O9—C41—C39	126.2 (4)
H17A—C17—H17B	109.5	O10—C41—C39	110.8 (4)
C19—C17—H17C	109.5	S3—C42—H42A	109.5
H17A—C17—H17C	109.5	S3—C42—H42B	109.5
H17B—C17—H17C	109.5	H42A—C42—H42B	109.5
C19—C18—H18A	109.5	S3—C42—H42C	109.5
C19—C18—H18B	109.5	H42A—C42—H42C	109.5
H18A—C18—H18B	109.5	H42B—C42—H42C	109.5
C19—C18—H18C	109.5	C44—C43—C48	119.4 (4)
H18A—C18—H18C	109.5	C44—C43—C38	119.3 (4)
H18B—C18—H18C	109.5	C48—C43—C38	121.3 (4)
C17—C19—C20	110.0 (5)	C45—C44—C43	120.9 (5)
C17—C19—C18	110.6 (5)	C45—C44—H44A	119.6
C20—C19—C18	110.3 (5)	C43—C44—H44A	119.6
C17—C19—H19A	108.6	C46—C45—C44	118.4 (5)
C20—C19—H19A	108.6	C46—C45—H45A	120.8
C18—C19—H19A	108.6	C44—C45—H45A	120.8
N3—C20—C21	121.0 (4)	C47—C46—C45	123.1 (5)
N3—C20—C19	115.7 (4)	C47—C46—F3	118.6 (5)
C21—C20—C19	123.3 (4)	C45—C46—F3	118.4 (5)
C22—C21—C20	118.4 (4)	C46—C47—C48	118.8 (5)
C22—C21—C25	122.9 (5)	C46—C47—H47A	120.6
C20—C21—C25	118.3 (4)	C48—C47—H47A	120.6
N4—C22—C21	120.5 (5)	C47—C48—C43	119.5 (5)
N4—C22—C27	113.9 (4)	C47—C48—H48A	120.2
C21—C22—C27	125.6 (4)	C43—C48—H48A	120.2
			
O3—S1—C7—N1	71.3 (4)	C19—C20—C21—C25	−7.2 (7)
O3—S1—C7—N2	−107.9 (4)	C20—C21—C22—N4	2.5 (7)
O4—S1—C7—N1	−58.1 (5)	C25—C21—C22—N4	−170.3 (5)
O4—S1—C7—N2	122.7 (4)	C20—C21—C22—C27	−177.7 (5)
C10—S1—C7—N1	−174.3 (4)	C25—C21—C22—C27	9.5 (8)
C10—S1—C7—N2	6.5 (5)	C22—C21—C25—O5	72.0 (7)
C8—O1—C9—O2	−2.9 (8)	C20—C21—C25—O5	−100.8 (7)
C8—O1—C9—C5	−178.3 (5)	C22—C21—C25—O6	−107.9 (5)
C7—N1—C4—C5	−2.5 (7)	C20—C21—C25—O6	79.3 (6)
C7—N1—C4—C3	178.7 (5)	N4—C22—C27—C28	−152.0 (5)
C4—N1—C7—N2	−1.2 (8)	C21—C22—C27—C28	28.2 (8)
C4—N1—C7—S1	179.7 (3)	N4—C22—C27—C32	24.6 (7)
C7—N2—C6—C5	4.4 (7)	C21—C22—C27—C32	−155.2 (5)
C7—N2—C6—C11	−174.7 (4)	C32—C27—C28—C29	0.1 (8)
C6—N2—C7—N1	0.3 (8)	C22—C27—C28—C29	176.7 (5)
C6—N2—C7—S1	179.3 (4)	C27—C28—C29—C30	−2.2 (9)
C2—C3—C4—N1	42.9 (7)	C28—C29—C30—F2	−177.7 (5)
C1—C3—C4—N1	−81.4 (6)	C28—C29—C30—C31	3.7 (10)
C2—C3—C4—C5	−135.9 (6)	F2—C30—C31—C32	178.4 (5)
C1—C3—C4—C5	99.8 (6)	C29—C30—C31—C32	−3.0 (10)
N1—C4—C5—C6	6.8 (7)	C30—C31—C32—C27	0.8 (10)
C3—C4—C5—C6	−174.4 (5)	C28—C27—C32—C31	0.5 (9)
N1—C4—C5—C9	−171.1 (5)	C22—C27—C32—C31	−176.2 (5)
C3—C4—C5—C9	7.7 (8)	O11—S3—C37—N5	36.4 (4)
C4—C5—C6—N2	−7.9 (7)	O11—S3—C37—N6	−147.9 (3)
C9—C5—C6—N2	170.0 (5)	O12—S3—C37—N5	−91.5 (4)
C4—C5—C6—C11	171.1 (5)	O12—S3—C37—N6	84.2 (4)
C9—C5—C6—C11	−11.0 (8)	C42—S3—C37—N5	153.1 (3)
C6—C5—C9—O2	−58.8 (7)	C42—S3—C37—N6	−31.2 (4)
C4—C5—C9—O2	119.1 (6)	C40—O10—C41—O9	3.8 (7)
C6—C5—C9—O1	116.8 (5)	C40—O10—C41—C39	−177.0 (4)
C4—C5—C9—O1	−65.3 (6)	C38—N5—C37—S3	174.5 (3)
N2—C6—C11—C16	−38.1 (7)	C38—N5—C37—N6	−0.5 (7)
C5—C6—C11—C16	142.9 (5)	C37—N5—C38—C39	−2.3 (6)
N2—C6—C11—C12	135.4 (5)	C37—N5—C38—C43	176.5 (4)
C5—C6—C11—C12	−43.6 (7)	C37—N6—C36—C39	−0.9 (6)
C16—C11—C12—C13	−1.3 (8)	C37—N6—C36—C35	−179.9 (4)
C6—C11—C12—C13	−174.9 (5)	C36—N6—C37—S3	−172.9 (3)
C11—C12—C13—C14	1.3 (8)	C36—N6—C37—N5	2.1 (7)
C12—C13—C14—C15	−2.0 (9)	C33—C35—C36—N6	−31.2 (6)
C12—C13—C14—F1	177.6 (5)	C34—C35—C36—N6	93.5 (5)
C13—C14—C15—C16	2.7 (10)	C33—C35—C36—C39	150.0 (5)
F1—C14—C15—C16	−176.9 (5)	C34—C35—C36—C39	−85.4 (6)
C14—C15—C16—C11	−2.6 (9)	N6—C36—C39—C38	−1.6 (6)
C12—C11—C16—C15	2.0 (8)	C35—C36—C39—C38	177.2 (4)
C6—C11—C16—C15	175.6 (5)	N6—C36—C39—C41	174.6 (4)
O7—S2—C23—N3	123.8 (4)	C35—C36—C39—C41	−6.6 (6)
O7—S2—C23—N4	−56.0 (5)	N5—C38—C39—C36	3.3 (6)
O8—S2—C23—N3	−107.6 (4)	C43—C38—C39—C36	−175.4 (4)
O8—S2—C23—N4	72.5 (4)	N5—C38—C39—C41	−172.8 (4)
C26—S2—C23—N3	8.6 (5)	C43—C38—C39—C41	8.5 (7)
C26—S2—C23—N4	−171.3 (4)	C38—C39—C41—O9	−128.5 (5)
C24—O6—C25—O5	0.4 (8)	C36—C39—C41—O9	55.4 (6)
C24—O6—C25—C21	−179.7 (4)	C38—C39—C41—O10	52.3 (5)
C23—N3—C20—C19	178.9 (4)	C36—C39—C41—O10	−123.8 (4)
C23—N3—C20—C21	0.9 (7)	N5—C38—C43—C44	35.1 (6)
C20—N3—C23—S2	−179.1 (4)	C39—C38—C43—C44	−146.2 (5)
C20—N3—C23—N4	0.7 (8)	N5—C38—C43—C48	−141.8 (4)
C23—N4—C22—C21	−1.1 (7)	C39—C38—C43—C48	37.0 (7)
C23—N4—C22—C27	179.1 (4)	C48—C43—C44—C45	−0.7 (8)
C22—N4—C23—S2	179.2 (3)	C38—C43—C44—C45	−177.6 (5)
C22—N4—C23—N3	−0.7 (8)	C44—C43—C48—C47	0.9 (7)
C17—C19—C20—N3	59.9 (6)	C38—C43—C48—C47	177.8 (4)
C18—C19—C20—N3	−62.3 (6)	C43—C44—C45—C46	0.1 (9)
C17—C19—C20—C21	−122.1 (5)	C44—C45—C46—C47	0.4 (10)
C18—C19—C20—C21	115.6 (6)	C44—C45—C46—F3	−179.3 (5)
N3—C20—C21—C22	−2.5 (7)	C45—C46—C47—C48	−0.1 (9)
C19—C20—C21—C22	179.7 (5)	F3—C46—C47—C48	179.6 (5)
N3—C20—C21—C25	170.7 (5)	C46—C47—C48—C43	−0.5 (8)

### Hydrogen-bond geometry (Å, °)

**Table:**

*D*—H···*A*	*D*—H	H···*A*	*D*···*A*	*D*—H···*A*
C3—H3B···O1	0.98	2.54	3.089 (7)	115
C17—H17C···F1	0.96	2.51	3.191 (8)	128
C19—H19A···O6	0.98	2.55	3.224 (7)	126
C28—H28A···O6	0.93	2.56	3.378 (6)	147
C8—H8B···O3^i^	0.96	2.48	3.373 (6)	155
C35—H35A···O12^ii^	0.98	2.57	3.484 (6)	154
C42—H42A···O9^iii^	0.96	2.43	3.229 (6)	141
C48—H48A···O12^iv^	0.93	2.42	3.224 (6)	146

Symmetry codes: (i) *x*, −*y*+1/2, *z*−1/2; (ii) −*x*+1, −*y*+2, −*z*; (iii) *x*, −*y*+3/2, *z*+1/2; (iv) *x*, −*y*+3/2, *z*−1/2.
